# Older adults produce more verbal false memories than younger adults: is it semantics or executive functioning?

**DOI:** 10.1007/s40520-024-02914-4

**Published:** 2025-03-17

**Authors:** Martina Cangelosi, Luca Rinaldi, Ton Dijkstra, Paola Palladino, Elena Cavallini

**Affiliations:** 1 University of Pavia, Cascina Cravino,Via Agostino Bassi, 21, Pavia, 27100 Italy; 2 Radboud University, Nijmegen, The Netherlands; 3 University of Foggia, Foggia, Italy

**Keywords:** Aging, False memory, DRM paradigm, Semantics, Executive functioning

## Abstract

**Background:**

A verbal false memory occurs when one remembers a word (called “critical lure”) as part of a previously presented list despite its absence. This phenomenon may be linked to the semantic associations of the critical lure with actual list items.

**Aims:**

We aimed to investigate the mechanisms behind the increase in verbal false memories with aging, evaluating whether this is due to a greater reliance on semantic processing or impaired executive functioning.

**Methods:**

We employed the Deese-Roediger-McDermott (DRM) paradigm, presenting DRM word lists to two age groups: young adults and older adults. In addition, participants completed the Hayling Sentence Completion Test to assess inhibition and the Backward Digit Span Task to evaluate working memory.

**Results:**

Our findings confirm that older adults experience more verbal false memories than younger participants. Results suggest that both semantic processing reliance and inhibition impairment contribute to the increase in false memories with aging, while working memory was not significantly related to false memory production.

**Discussion:**

Older adults’ increased susceptibility to false memories appears to arise from an interplay between enhanced semantic reliance and inhibition deficits.

**Conclusions:**

This study proposes a novel integration of semantic and executive mechanisms underlying the observed increase in false memories during aging, with inhibition playing an unexpected role in enhancing false memory susceptibility.

## Highlights

Older adults produce more verbal false memories than younger adults.

Verbal false memories in younger adults are due to semantic spreading activation.

The DRM paradigm provides a valid index of semantic processing in younger adults.

Older adults’ increase in verbal false memories is due both to semantics and executive functioning.

## Introduction

False memories occur when individuals believe they remember an event or detail that never happened. A widely used method to study this phenomenon is the Deese-Roediger-McDermott (DRM) paradigm [1, 2]. In the DRM task, participants are presented with a list of semantically related words and later asked to recall or recognize these words. This often leads to the false recall of a related but non-present word known as the “critical lure” (e.g., recalling “water” after a list of words related to it).

False memories are generally explained by two main theories: Fuzzy Trace Theory (FTT) and Spreading Activation Theory (SAT). FTT suggests that memory involves two types of processing: verbatim (specific details) and gist (general meaning) [3]. False memories occur when the gist of an event is remembered but the specific details are not [4]. This dual-process model has been extensively developed and applied in memory development and false memory research [5]. SAT posits that activating a concept spreads to related concepts, leading to false memories due to the activation of semantically related words [2].

Research shows that younger adults and children are prone to false memories due to semantic processing [6, 7, 8, 9], but the mechanism in older adults remains debated [10, 11]. Older adults tend to produce more verbal false memories than younger individuals [12, 13, 14, 15], possibly due to greater reliance on gist processing or declining executive function.

This study aims to determine whether the increase in false memories with age results primarily from greater reliance on semantic processing or decreased executive functioning. We propose a new hypothesis integrating both explanations.

### False memories’ increase with age is related to changes in semantic processing

Regarding the semantic account, some authors argue that as adults age, they rely more on semantic elaboration when memorizing DRM lists. Tun et al. [16], based on three DRM-based experiments, proposed that older adults’ increased false memory production stems from greater reliance on gist strategies. Similarly, Dennis, Kim, and Cabeza [12] found older adults prefer gist-based over item-specific encoding. Neural evidence shows age-related activation of the superior temporal gyrus (STG), responsible for semantic processing [17], linked to both true and false retrieval. In a later study [18], they suggested older adults’ decline in true memory retrieval might reflect hippocampal changes [19], while enhanced gist reliance could involve the lateral temporal cortex, crucial for language comprehension [20, 21].

These findings align with studies on semantic processing during aging. Birren [22] concluded older adults rely heavily on semantic knowledge for encoding and retrieval in laboratory and environmental contexts. Recent research highlights the interconnectedness of semantic cognition with neural and cognitive networks in aging, emphasizing lifelong education and adaptability [23]. Some studies report better semantic performance in older adults. For instance, Zhuang et al. [24] found older adults had higher accuracy rates in a semantic similarity judgment task than younger adults.

Taylor and Burke [25] showed only semantically related distractors interfered with older adults in a picture-naming task, suggesting semantic memory is preserved with age, unlike episodic memory. Wu and Hoffman [26], using fMRI, found preserved semantic processing in aging, with similar activations in central semantic network areas across age groups. Despite neural changes, older adults maintain strong semantic performance through compensatory strategies involving additional brain regions.

However, some studies challenge this view. Au et al. [27] and Barresi [28] reported semantic-lexical memory decline starting around age 70. Verhaegen and Poncelet [17] found older adults performed worse than younger controls on the PPTT [29] and a synonym judgment task [30], assessing semantic knowledge. Zhu et al. [31] suggested semantic performance might depend on functions like processing speed. Thus, semantic increases in aging are not universal and interact with other cognitive domains [32, 33].

Other evidence supports the second account of age-related false memories, attributing them to deteriorating executive functioning (e.g [34]).

### False memories’ increase with age is related to changes in executive functioning

Askey and Playfoot [35] explicitly contrasted a semantic and an inhibition hypothesis for the increase of false memories with age. First, they considered the Transmission Deficit Hypothesis, which suggests that there is a reduction in the strength of links in the semantic network due to aging. Second, the Inhibitory Deficit Hypothesis attributes the increase in false memory in aging to inhibition mechanisms that become less efficient. Based on the results of their experiments involving a manipulation of list length, the authors suggested that the Inhibitory Deficit Hypothesis provides a better explanation for the formation of false memories in aging. This theory posits that the semantic network in older adults’ functions similarly to that in younger adults, but aging reduces the efficiency of ignoring unnecessary nodes. This model is supported by other studies present in the literature (e.g [8]).

In addition, Askey and Playfoot [35] criticized Tun et al.’s [16] view of semantic enhancement in aging, arguing that if there were a genuine improvement in the semantic field, it should result not only in an increase in false memories but also in true memories, due to a general improvement in the task. However, their study did not show this pattern: they found an increase in verbal false memory alongside a reduction in true memory. Their results suggest an impaired performance, rather than an enhanced one.

The debate about the reason why older adults produce more verbal false memories is still ongoing (e.g [36, 10, 37]).and it may be a further lens through which to read the complex interplay of changes that the older adult goes through.

## Aim of the study

The primary aim of this study is to systematically test semantic and executive control theories in the increase in verbal false memories observed with aging.

Based on the existing literature and the aim of this study, we propose the following hypotheses:

### Hypothesis 1

The semantic similarity of the words in the DRM lists will be positively correlated with the number of false memories in both age groups, with a more pronounced effect in older adults.

### Hypothesis 2

Executive functioning, specifically inhibition and working memory, will have a differential impact on false memory production based on age. We expect that older adults will demonstrate greater difficulty inhibiting strongly semantically associated stimuli, leading to increased reliance on semantic processing, despite a decline in executive functioning.

### Hypothesis 3

Older adults who perform better on inhibition tasks will still produce more semantically related false memories, suggesting that effective executive functioning is necessary to manage the semantic demands of the DRM task.

## Method^1^

### Participants

We included a total of 56 Italian monolingual participants, divided into two groups: one with 31 young participants (Mean Age = 28.69, Range = 25–32, Female participants = 64.52%) and one with 25 older adults (Mean Age = 68.74, Range = 65–76, Female participants = 36%).

As an inclusion criterion, participants in the younger group had to be between 20 and 35 years old at the time of testing, and participants in the older adult group had to be between 65 and 80 years old. The older group was administered the Mini Mental State Examination (MMSE) [38], which assesses a person’s neuro-cognitive and functional state. Only participants who passed the 24-point cutoff, commonly used as a benchmark to distinguish healthy aging from pathological aging, were included in the older adult group. Since none of the older participants scored below the threshold, all were included in the sample.

### Materials

All materials were presented, compiled, and collected via computer. The sessions took place in quiet environments, without distractions, and participants were instructed to use devices with stable Internet connections. An experimenter monitored each session via Skype to intervene in case of technical problems or distractions. During each phase, participants were asked to confirm their understanding of the instructions, minimizing the risk of errors related to the telematic method.

#### DRM task

We started from a pool of 32 DRM lists, each containing 15 words. These lists were adapted to Italian from materials developed and validated by Brainerd and colleagues (Cornell/Cortland Emotional Lists, CEL Lists) [39]. The adaptation process involved translating the lists into Italian and ensuring cultural and semantic equivalence. Words that were not directly translatable or lacked a clear semantic association in the Italian context were replaced with culturally appropriate alternatives. For example, the word “needle” (originally highly associated with “thread” in English) was replaced with “ago” to maintain the semantic relationship within the Italian lexicon.

The validation of the adapted materials involved a pilot study with 20 healthy older adults (Mean age: 74) and 20 young adults (Mean age: 26.5), who did not participate in the main study. Word frequency was evaluated using the CoLFIS Lexical Database [40], ensuring that words were common and familiar to Italian speakers. The Backward Associative Strength (BAS) index, measuring how strongly each word in the list is associated with the critical lure [41], was assessed using a 7-point Likert scale (1 = not at all associated to 7 = very associated). BAS values from the Italian lexicon showed comparable patterns to those in the original CEL Lists, suggesting that the adapted materials preserved relational structures despite linguistic differences.

Stimulus words were ordered from most to least associated based on the BAS derived from our pilot study [10]. A key comparison showed that while some item orders shifted slightly due to associative norm variations [42], the critical lures elicited similarly high associations in both the original and adapted lists. During the coding phase, 12 of the 32 adapted lists were presented to participants, each containing the first 10 words most strongly associated with the semantically related but non-present critical lure.

In the recognition phase, for each list, we presented 3 words shown in the coding phase (9 in total), the critical lure for each list (12 in total), and 1 semantically related distractor (the 12th word from the original list of 15). The semantically related distractors were less correlated in meaning than the lures but still within the same semantic sphere (e.g., “injection” from the list where “needle” was the critical lure). Finally, we included 2 non-semantically related distractors, selected from the 20 remaining rejected lists of the 32 originally adapted (6 in total) (e.g., “spider”).

#### Semantic similarity index

We computed a semantic similarity index for the three categories of new words presented during the recognition phase: critical lures, semantically related distractors, and non-semantically related distractors. This index was calculated using distributional semantic models (DSMs), which represent word meanings as high-dimensional numerical vectors extracted from extensive natural language data [43]. DSMs meet the criteria for psychological models of semantic representation and memory structure [44].

Vector representations for these words were extracted from the semantic space WEISS1-Italian forms, based on the Continuous Bag of Words (CBOW) model [45], trained on the Italian-text corpus ItWac. Parameters for vectorization were set to 400 dimensions with a 9-word window size, meaning predictions considered a target word surrounded by 4 words on each side. Negative sampling was set at k = 10, referring to distinguishing target words from noise distribution draws [33], and subsampling was set at t = 1e-5 to reduce the impact of frequent uninformative words.

Pairwise comparisons of words in this semantic space were conducted. A semantic similarity index (SSim) was calculated for each pair as the cosine of the vector angle representing the distance between the words. Higher cosine values indicated greater semantic proximity. Each index was subtracted from 1 to rearrange the values on a proximity scale and weighted by raw frequency, obtained from the SUBTLEX-IT database [36], following Gatti et al. [28].

**Fig. 1:**
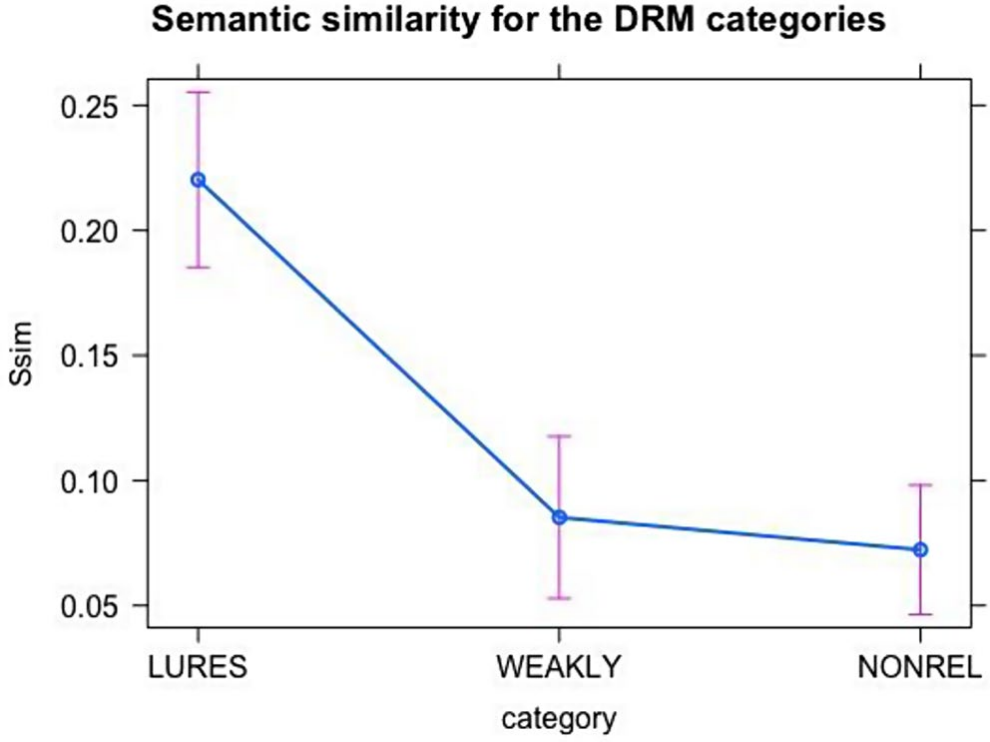
Semantic similarity for the DRM categories of critical lures, weakly related and unrelated words

To confirm that semantic similarity scores captured differences in relatedness with studied words, a one-way ANOVA was conducted with the semantic similarity index as the dependent variable and the three distractor categories as the independent variable. The effect of word category was significant (*p* <.01, F(2, 85) = 25.58, 95% C.I.). Pairwise comparisons showed larger semantic similarity for lures (Mean = 0.220, SD = 0.017, from estimated marginal means), followed by semantically related distractors (Mean = 0.085, SD = 0.016), and non-semantically related distractors (Mean = 0.072, SD = 0.013) [44] (see Fig. 1).

The impact of semantic similarity on false memories was further tested with distractor categorization as a covariate. This effect was significant (*p* <.01), confirming the semantic similarity index provided additional insight beyond the tripartite distractor categorization [46].

#### Cognitive measures

##### Inhibition task^2^

We applied the Hayling Sentence Completion Test [47]. The test consists of two sections, each composed of 15 sentences with the last word missing. In the first section (Section A), the participant is asked to complete a sentence with the correct word according to the sentence context. An example of a sentence from Section A with correct completion is: “On a motorcycle you must always wear…”– helmet.

In the second section (Section B), the participant is asked to complete the sentence with a word that is not related in terms of meaning to the context of the sentence, but which agrees in gender and number with the preceding article. An example of a sentence from Section B and its correct completion is: “Bees produce…”– traffic.

Scoring was obtained by considering accuracy for each sentence, following the guidelines included in the task. At the end of the two sections, a general index was obtained by subtracting the score obtained in Section B from the score obtained in Section A.

#### Working memory task

The Backward Digit-Span Task was administered [50]. The task was administered as a measure of working memory. The participant is verbally presented with series of numbers, divided into blocks of 3 sequences each. Within each block, the sequences have the same length. Blocks are divided considering the gradually increasing length of the sequences they contain.

Participants repeat each sequence, at the end of the oral presentation of the same sequence, in reverse order compared to how it was read (e.g., the experimenter reads: “5-7-9”; the participant replies: “9-7-5”).

The task ends when the participant misses two out of three sequences within a block. The quantity of numbers of the last correctly executed block constitutes the score obtained in the task. For example, participants will obtain a score of 4 if they miss two sequences in the 5-digit block.

### Procedure

The test involved a single one-hour session per participant, conducted remotely via Skype due to the Sars-Cov-2 (Covid-19) pandemic. Participants received a Google Forms link via email to provide consent and share personal data, including age, education, and profession. Profession was assessed as an index of socio-economic status using an adapted Hollingshead Four Factor Index questionnaire [14]. Participants shared their screens to review the consent form and were guided in completing the questions.

Before the experiment, the Mini Mental State Examination (MMSE) was administered [33]. Questions were read aloud, and responses were recorded by the experimenter. Participants viewed an associated drawing on screen, copied it onto paper, and displayed it for the experimenter. The drawing was saved via screenshot.

The four tasks (three control and one experimental) were administered in randomized order. A ten-minute break was scheduled midway through the session.

### DRM task

The experimental task was built using the OpenSesame 2.0 program [51] and presented to the participants through the Just Another Tool for Online Studies (JATOS) platform [52], which allowed sharing the task via a link. The procedure adopted for administration was the same as the one proposed by the authors of the original lists [39]. During the coding phase, three lists of items appeared on the screen, with one word at a time, and with a 10-second gap between one list and the next. The stimuli were presented in “mono” font, size 18, centered on the screen.

Participants were instructed to memorize as many words as possible because they would later be asked to recognize them among other words not previously seen. Following the first three item lists, the first recognition task was presented. Participants were instructed to press the space bar every time they recognized one of the previously memorized words, trying to be as fast and accurate as possible. Between the coding phase and the recognition task, a 30-second pause was provided, during which generic questions relating to perceived fatigue or difficulty of the task were asked. The aim of these questions was to distract participants from the words they had just memorized. This procedure was repeated four times, each time with different lists.

#### Executive functions

The inhibition task and the working memory task were read aloud by the experimenter to participants. Answers were recorded and written directly by the experimenter while participants pronounced them.

## Results

For all analyses conducted below, we used the lme4 package and the lmerTest package in RStudio 1.0.153 [53]. This allowed us to build generalized linear mixed-effects models using the ‘glmer’ function.

We began by testing whether aging was associated with an increased number of verbal false responses to gather support for our confirmative hypothesis. For this purpose, we ran a linear mixed model with the number of false memories (i.e., the number of new words that were recognized as already seen) as the dependent variable, participant group (young vs. old) as the dichotomous independent variable, and participants and words as random intercepts.

To answer our main research question, we performed a model selection using the MuMIn R package, with the function ‘dredge’ [54]. This procedure selects the best-fitting model (i.e., the one with the lowest Akaike information criterion, AIC) [21], which provides an estimation of the quality of the model.

We also checked whether one or more models had a ΔAIC < 2 compared to the best model identified by the dredge selection, as models in this AIC range can be considered equivalent in explaining participants’ performance [21]. We found only one model with ΔAIC = 1.37, in which the interaction between group and span was dropped. However, in the full model reported in the Results section, the interaction between group and span was not significant. Given this, we inserted false memory as the dependent variable, with group as the main effect, in interaction with the semantic similarity of the items, inhibition (measured by the Hayling Test [47]), and working memory capacity (measured by the Backward Span Test [50]) (see Tables 1 and 2 for separate correlations between the covariates in the two groups).

**Table 1:** Correlation matrix between cognitive measures in the older adults group

	SSim	inhibition	Working memory
SSim	X	0.00	0.00
inhibition	0.0	X	− 0.08
Working memory	0.0	− 0.08	X

**Table 2:** Correlation matrix between cognitive measures in the younger adults group

	SSim	inhibition	Working memory
SSim	X	0.00	0.00
inhibition	0.0	X	− 0.02
Working memory	0.0	− 0.02	X

**Fig. 2:**
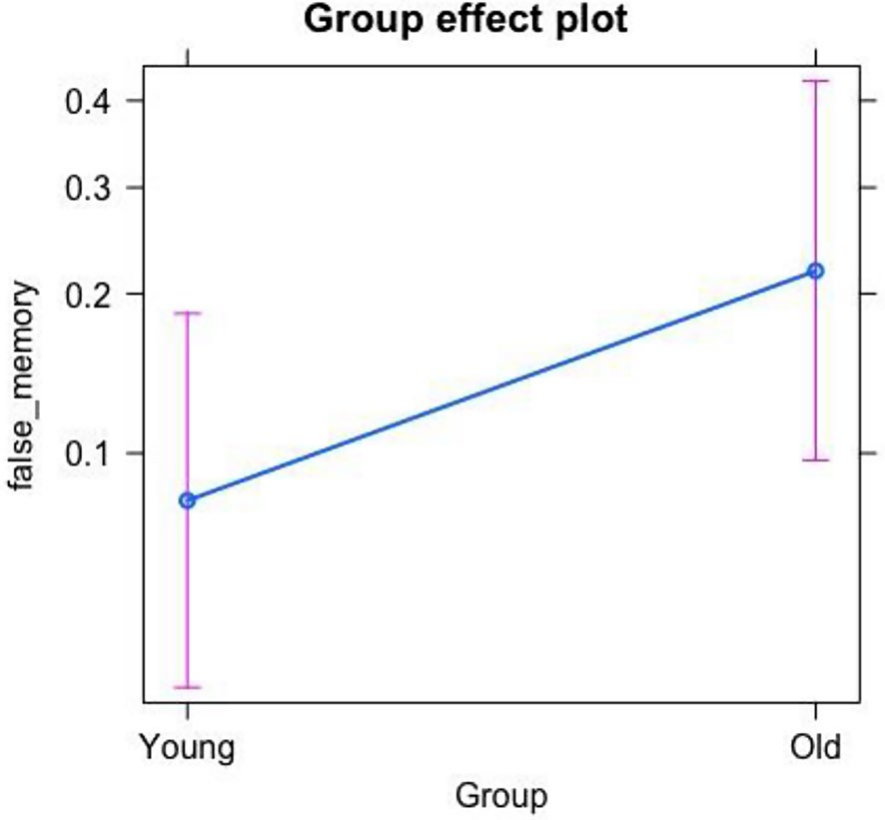
False memory effect between the two groups

Concerning the first model (see Fig. 2), built to test our confirmative hypothesis, the older adults’ group was found to produce a significantly higher average of false memories relative to the young group (*p* =.031, Estimate = 1.167).

**Fig. 3:**
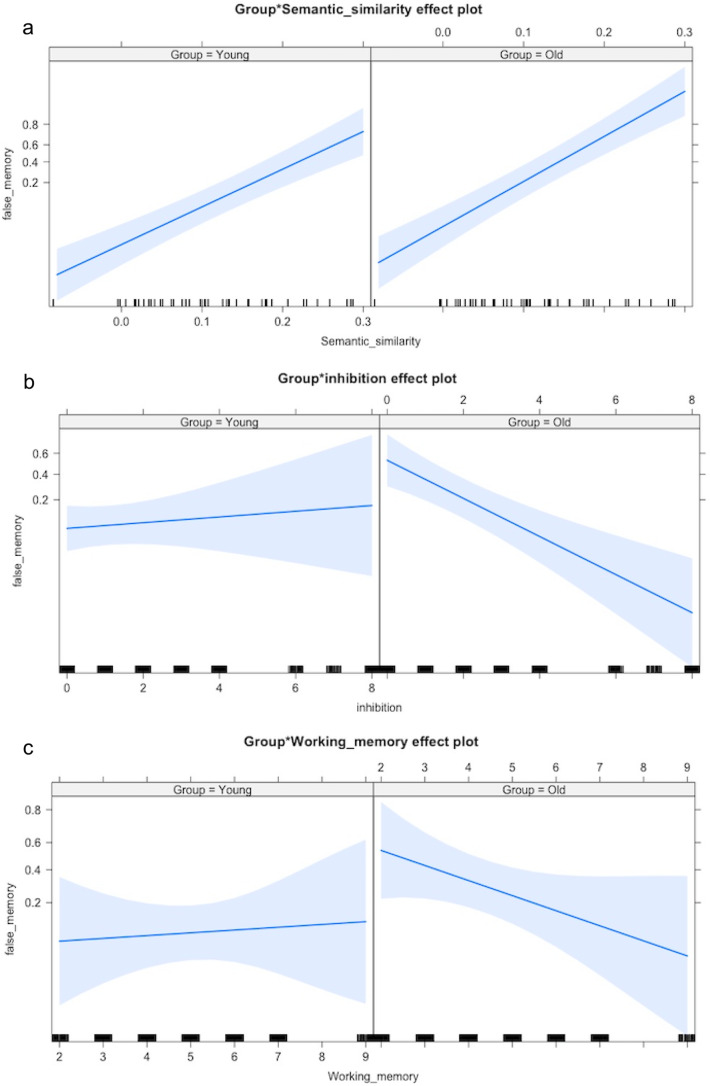
Linear mixed model results

For the second model, aimed at answering our main research question, semantic similarity and group proved both to be significant (respectively with *p* <.001, t (46) = 9.130, Estimate = 17.891, χ2 (1) = 56.395 and *p* =.020, Estimate = 4.749, χ2 (1) = 5.458). Inhibition significantly predicted the production of false memories in interaction with the older group (*p* <.001, Estimate = − 0.844, χ2 (1) = 10.912). This demonstrates that a better performance in the inhibition task is accompanied by an increased production of false memories (See Fig. 3).

The interaction between semantic similarity and the older group showed a trend marginally significant (*p* =.051, Estimate = 3.450, χ2 (1) = 3.808). In contrast, working memory was not significant in predicting false memory performance in the overall sample (*p* =.780, Estimate = 0.083, χ2 (1) = 0.078), nor in interaction with the older group (*p =*.173, Estimate = − 0.533, χ2 (1) = 1.856) (see Fig. 3 for the interaction effects).

Furthermore, we ran the same analyses after dividing (instead of multiplicating) the main effect (i.e., the variable “group”) in terms of the other variables. The main effect with Semantic Similarity was significant in both groups (Older adults: *p* <.001, Estimate = 21.390, χ2 (2) = 77.591; Younger adults: *p* <.001, Estimate = 17.891, χ2 (2) = 77.591). The interaction of group with the inhibition was significant only for the older adults (*p* <.001, Estimate = − 0.734, χ2 (2) = 20.001), but not for the younger adults (*p* =.571, Estimate = 0.110, χ2 (2) = 20.001). The working memory measure did not significantly interact with either group (older adults: *p* =.077, Estimate = − 0.450, χ2 (2) = 3.210; younger adults: *p* =.780, Estimate = 0.083, χ2 (2) = 3.210).

## Discussion

In this study, we examined whether the increased occurrence of false memories in older participants was due to greater reliance on semantic processing [12, 55, 25] or executive functioning impairment [27, 28, 38]. Instead of contrasting these aspects, we hypothesized that both contribute. To examine semantic factors, we analyzed the similarity between studied and new words in the DRM task, predicting performance based on this index. To assess executive factors, we evaluated inhibition and working memory, known to influence false memory production [56, 10, 57, 58].

We first confirmed that older adults produced more verbal false memories than younger participants, consistent with prior evidence [13, 57, 8].

For semantics, we hypothesized:

- Higher semantic similarity in DRM lists correlates with more false memories [6, 59].
- Semantic effects on verbal false memories would be stronger in older adults [22, 25].

Results indicated semantic similarity significantly influenced false memory production across the general population, consistent with previous literature [6]. Although the interaction between semantic similarity and the older group approached significance, analyses revealed no clear pattern. Critical lures were most falsely recognized, followed by semantically related distractors and unrelated distractors.

The marginal interaction suggests older adults may be more influenced by semantic relationships between studied and new items. A larger sample might reveal significant interactions, reinforcing semantic similarity as a key driver of false memory production in older adults.

These findings align with existing literature [60] but do not solely attribute increased false memories in older adults to semantics.

For executive functioning, we expected its impact to differ across groups. Executive functioning, known to decline with age [16, 17], was hypothesized to influence false memory performance more in older adults.

In older adults, inhibition played a role, while working memory did not significantly affect false memory production. Unexpectedly, better inhibition correlated with more false memories, consistent with prior findings [13]. Although older adults performed worse overall than younger adults (see Table 3), those with better inhibition produced more semantic false memories [61].

**Table 3:** Group differences in cognitive measures

	Older adults	Younger adults	*p* value
Inhibition (Hayling Test)	2.185	1.103	< 0.01
Working Memory (Backward Span)	4.407	5.034	< 0.01

This finding challenges the assumption that better inhibition reduces false memories. The Hayling Test, traditionally measuring inhibitory control, also assesses semantic control abilities. Semantic control, or the ability to manipulate meaningful information based on context [62, 63], correlates strongly with semantic representation [64]. This could explain why participants with better inhibition scores produced more semantically related false memories.

Enhanced semantic control in older adults may activate semantic networks, facilitating gist-based processing—a known trigger for false memories [5]. Neuroimaging studies show older adults with greater activation in semantic processing regions, such as the lateral temporal cortex, are more likely to produce false memories [18]. Thus, better semantic control may inadvertently increase susceptibility to false memories by enhancing retrieval of semantically related concepts.

Alternatively, this result may reflect compensatory mechanisms. Older adults with intact executive functioning may rely more on semantic processing for memory retrieval, amplifying the influence of semantic similarity on false memory production. From this perspective, semantic activation in aging is not merely a consequence of impaired executive functioning but a compensatory resource activated effectively when cognitive functions are intact.

These findings underscore the complex relationship between inhibition and false memories, particularly in aging populations [55, 23]. They highlight the multidimensional nature of cognitive measures, where tasks like the Hayling Test reflect overlapping processes such as inhibition, semantic control, and memory integration [62, 64]. While lacking a purely inhibition-focused measure is a limitation, it suggests semantic control could bridge the theories of semantic reliance and inhibitory deficits [23]. Neuroimaging studies [62, 55] support this integration, showing dynamic interactions between semantic control and executive processes. Chamberlain et al. [32] found that encoding-retrieval similarity and cognitive mechanisms beyond semantics influence age-related differences in false memory. This underscores the importance of integrating semantic and executive factors in aging research.

To summarize, while inhibition deficits are commonly linked to false memory production, our findings suggest that in older adults, better inhibition—as indexed by tasks involving semantic control—may paradoxically enhance false memory susceptibility by facilitating gist-based retrieval [5, 4]. This interpretation bridges semantic and executive accounts of age-related false memories, supporting an integrative model where these factors are complementary rather than competing [62, 23]. Future studies should include domain-specific measures of inhibition and semantic control to clarify their contributions to false memory formation [18, 15, 31].

Our study provides new insights into how semantic processing and inhibitory control interact in false memory production in older adults. Aging enhances semantic competence, functioning as a compensatory mechanism for memory deficits [22], but executive declines, including inhibitory control, distort memory accuracy [35]. Our analysis suggests verbal false memories arise from a dynamic interaction between enhanced semantics and inhibitory deficits [65]. This integrative approach extends existing research, proposing a model where these factors complement each other in explaining increased false memories with age [12, 13, 14]. Furthermore, our findings imply false memories reflect not only deterioration but also functional reorganization of cognitive resources.

## Conclusions

In the present study, we hypothesized that semantics would influence older adults’ verbal false memories more than younger adults. During aging, semantic knowledge remains stable or even improves [22], becoming the primary reliance as general memory declines. However, executive functioning is often compromised in older adults [22, 64, 66], leading us to expect greater difficulty inhibiting semantically related items.

Our results partially confirmed these hypotheses. Both semantics and executive functioning contribute to the higher number of verbal false memories in older adults, but executive functioning played an unexpected role. Older adults who performed better on the inhibition task also produced more false memories. As the inhibition task is verbal and assesses semantic control, this may indicate that intact executive functioning is crucial for activating semantic knowledge and networks involved in the DRM recognition task. This aligns with Feng [23], who highlighted the role of interconnected semantic cognition and other cognitive processes in maintaining semantic performance despite neural changes with age.

Thus, semantic activation in aging is not merely a passive consequence of task difficulty and compromised functions. Instead, it serves as a resource utilized more effectively when executive functioning is intact. Future studies should explore this further using varied inhibition measures to clarify its role.

## Data Availability

The data are available upon request from the corresponding author.

## Footnotes

1 The study has been approved by the ethical committee of the University of Pavia, and it has been assigned with the number 34/19 for the practice

2 We chose this test in light of its established reliability and validity in assessing executive functions, particularly inhibitory control. Studies like that of [48] provided normative data for the Italian population, confirming the effectiveness of the test in measuring verbal inhibition in different age groups. Furthermore, it is sensitive to a wide range of inhibitory abilities, making it suitable for studying individual variations even in samples with different characteristics, such as the older adults (e.g [49]). It is simple to administer and takes approximately five minutes, reducing participant fatigue, which is crucial when working with older populations.Although HSCT includes a semantic component (see the “discussion” section where the limitations of the study are discussed in more depth), this makes it relevant to our study on verbal false memories, as it allows us to examine the interaction between inhibitory control and semantic processing. The test’s ability to assess inhibition in verbal context makes it particularly useful for investigating how these processes interact in the formation of false memories (e.g [11]).
